# Functional Microgel Enables Effective Delivery and Colonization of Probiotics for Treating Colitis

**DOI:** 10.1021/acscentsci.3c00773

**Published:** 2023-07-13

**Authors:** Bingyu Chen, Wendan Pu, Jianxiang Zhang

**Affiliations:** † Women and Children’s Hospital, Chongqing Medical University, Chongqing 401147, China; ‡ Department of Pharmaceutics, College of Pharmacy, Third Military Medical University (Army Medical University), Chongqing 400038, China

Over 2000 years ago, the Greek physician Hippocrates of Kos, lauded as the father of western medicine, proclaimed that “All disease begins in the gut”. The gut microbiome exerts pivotal roles in health and diseases, by regulating critical biological processes in metabolism, inflammation, and immunity. Dysbiosis of the gut microbiota is involved in the pathogenesis of numerous diseases, such as obesity, diabetes, inflammatory bowel disease (IBD), liver diseases, respiratory diseases, cardiovascular diseases, autism, anxiety, schizophrenia, Parkinson’s disease, Alzheimer’s disease, infectious diseases, and cancers.^1−4^ Accordingly, regulation of the homeostasis of the gut microbiome represents a promising strategy for the management of many human diseases. Both preclinical and clinical studies have demonstrated the effectiveness of microbiota manipulation in the treatment of IBD and neurodegenerative diseases.^4−6^ In particular, increasing attention has been paid to oral probiotic therapies for treating or curing colitis. Tablets, capsules, and powders of typical probiotic strains, such as *Bifidobacteria* and *Lactobacilli* that have long been used in the clinic, are available on the market; however, traditional formulations of such probiotics show poor stability and low bioavailability, thus limiting their therapeutic effects.

**Figure 1:**
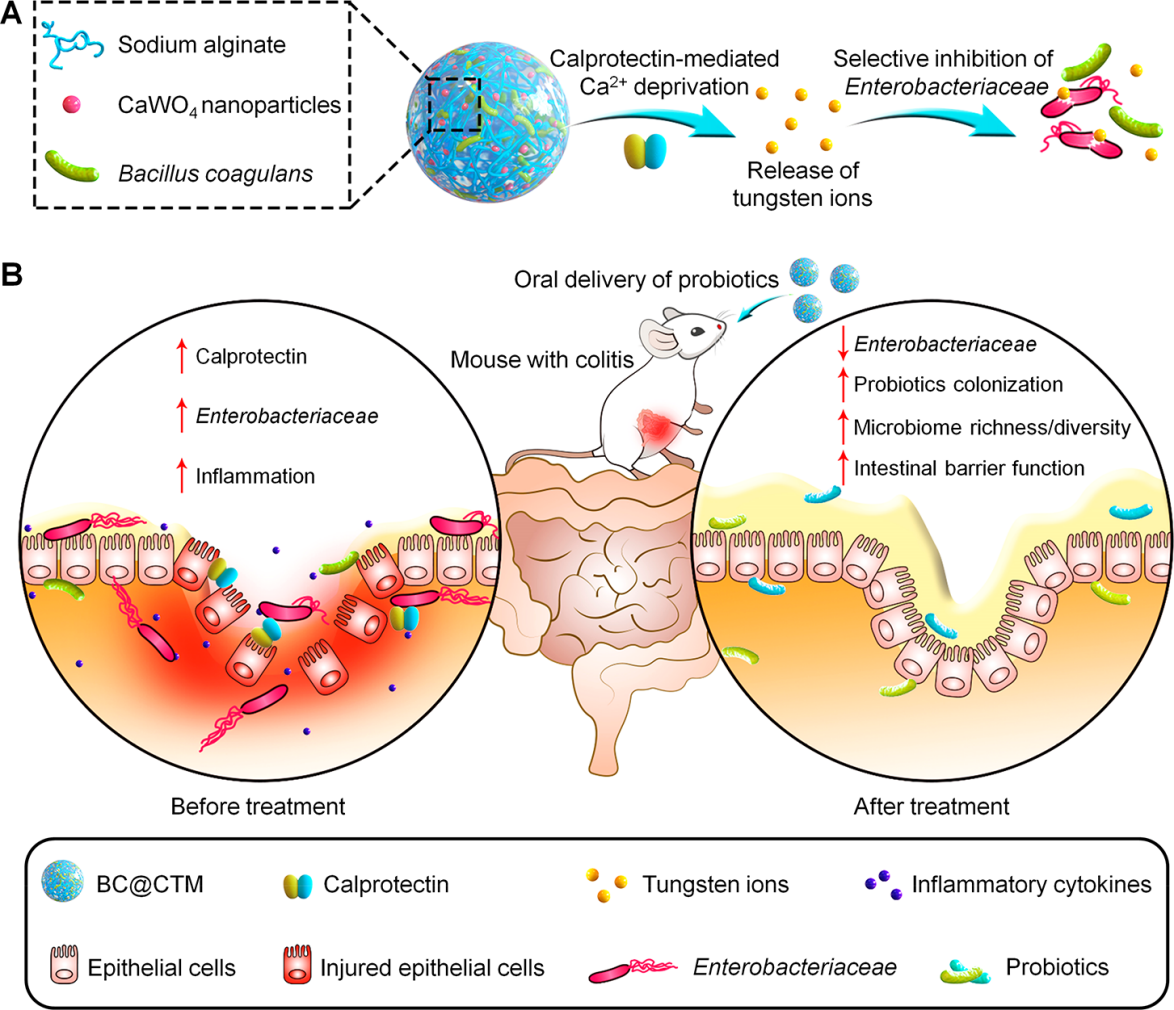
(A) Engineering of a calcium tungstate microgel (CTM) for the oral delivery of probiotics. (B) Oral delivery of *Bacillus coagulans*-containing CTM (BC@CTM) shows desirable therapeutic effects on colitis in mice by selectively suppressing abnormally expanded *Enterobacteriaceae* and promoting probiotic colonization.

Most recently, different surface engineering approaches and advanced delivery systems have been developed for probiotics to improve their stability in the gastrointestinal tract, increase oral delivery efficiency, and potentiate *in vivo* efficacies. However, therapeutic benefits of probiotic therapies remain to be improved, with respect to clinical translation. In this issue of *ACS Central Science*, Zhang, Liu, Shi, and co-workers rationally designed and constructed a multifunctional calcium tungstate microgel (CTM)-based system for oral probiotic delivery.^1^ CTM was formulated by the Ca^2+^-mediated cross-linking of sodium alginate and sodium tungstate in aqueous solution, giving rise to calcium tungstate (CaWO_4_) nanoparticle-loaded alginate microgels (Figure 1A). Using this facile and eco-friendly approach, probiotics can be conveniently packaged into CTM without affecting their biological performances. CTM was stable in simulated gastric fluid, while it gradually eroded upon incubation in simulated intestinal fluid, leading to the release of CaWO_4_ nanoparticles. More importantly, the erosion of CTM and release of CaWO_4_ nanoparticles and tungsten ions can be effectively triggered by calprotectin (CP), a highly expressed protein at colitis sites, due to strong interactions between CP and Ca^2+^. Released tungsten can inhibit the growth of *Enterobacteriaceae* by attenuating *Enterobacteriaceae*-dependent molybdenum enzyme activity. Since the growth of probiotics is independent of molybdenum enzymes, tungsten has no significant effects on probiotics loaded in CTM. Consequently, CTM can selectively suppress the growth of *Enterobacteriaceae* in colitis, thus disrupting the ecological niche occupied by pathogenic bacteria and promoting probiotic colonization. Using *Bacillus coagulans* (BC) as a candidate probiotic, the authors demonstrated that orally delivered BC-containing CTM (i.e., BC@CTM) effectively inhibited the proliferation of harmful bacteria, facilitated probiotic colonization, and restored gut microbiota homeostasis in mice with dextran sulfate sodium (DSS)-induced colitis (Figure 1B). Moreover, treatment with BC@CTM significantly alleviated local oxidative stress and inflammation and restored the intestinal barrier function. These beneficial effects collectively contribute to desirable therapeutic outcomes of BC@CTM on DSS-induced colitis in mice.

Previously, various types of delivery systems and bacterial functionalization approaches, such as biofilm-based encapsulation, hydrogels, and chemical/physical coating via different materials, have been established for the oral delivery of probiotics.^7−9^ Despite their effectiveness in protecting probiotics from damage in the gastrointestinal tract under physiological conditions, these strategies cannot efficiently and precisely normalize the pathological microenvironment, in particular, the proliferation and abnormal colonization of pathogenic bacteria that may severely inhibit probiotic colonization, thereby resulting in impaired pharmacological effects. Whereas the use of antibiotics enables intestinal decolonization of harmful bacteria, it also eliminates beneficial microbes and simultaneously causes antibiotic resistance. As a proof of concept, this work provides an intriguing and facile delivery strategy to improve oral delivery and colonization of probiotics under inflammatory conditions simply by using a rationally engineered microgel platform capable of selectively breaking the ecological niche of pathogenic bacteria.

As for the limitations of this study, only one mouse model of colitis and one probiotic were used. Future translation studies should validate the proposed strategy in other animal models of colitis and using different probiotic strains. In addition, gut microbiota-derived metabolites, such as trimethylamine/trimethylamine-*N*-oxide, secondary bile acids, and short-chain fatty acids, should be included in future analyses, in view of their critical effects on host immune responses and immune-related inflammatory diseases. Both the formulations and treatment regimens remain to be optimized to afford maximized efficacies and minimized side effects. There are also safety concerns regarding the use of high doses of tungsten and/or long-term exposure to tungsten. In addition to its direct toxicities to specific organs/tissues, emerging evidence suggests that tungsten can amplify the effects of other toxicants and endogenous/exogenous stressors.^10^ Finally, it would be interesting to explore the effectiveness of the proposed strategy for the treatment of other relevant diseases, such as periodontitis, liver diseases (e.g., nonalcoholic fatty liver disease and nonalcoholic steatohepatitis), common lung diseases (such as asthma and chronic obstructive pulmonary disease), neurological disorders (Alzheimer’s disease, Parkinson’s disease, stroke, etc.), and vaginal diseases, considering the presence of the microbiota in other tissues such as the oral cavity, respiratory tract, and vagina as well as the well-recognized gut–liver, gut–lung, and gut–brain axes.^2,4,6,11^
